# Genotype-Related Differences in the Phenolic Compound Profile and Antioxidant Activity of Extracts from Olive (*Olea europaea* L.) Leaves

**DOI:** 10.3390/molecules24061130

**Published:** 2019-03-21

**Authors:** Hakime Hülya Orak, Magdalena Karamać, Ryszard Amarowicz, Adnan Orak, Kamila Penkacik

**Affiliations:** 1 Department of Food Technology, Vocational School of Technical Sciences, Namik Kemal University, 59030 Tekirdağ, Turkey; horak@nku.edu.tr; 2 Department of Chemical and Physical Properties of Food, Institute of Animal Reproduction and Food Research, Polish Academy of Sciences, Tuwima 10, 10-748 Olsztyn, Poland; r.amarowicz@pan.olsztyn.pl (R.A.); k.penkacik@pan.olsztyn.pl (K.P.); 3 Field Crops Department, Agricultural Faculty, Namik Kemal University, 59030 Tekirdağ, Turkey; aorak@nku.edu.tr

**Keywords:** olive leaf extract, phenolic profile, antioxidant activity, GGE biplot analysis, cluster analysis, olive genotypes

## Abstract

The phenolic compound contents and antioxidant activities of the leaf extracts of nine olive genotypes were determined, and the obtained data were analysed using chemometric techniques. In the crude extracts, 12 compounds belonging to the secoiridoids, phenylethanoids, and flavonoids were identified. Oleuropein was the primary component for all genotypes, exhibiting a content of 21.0 to 98.0 mg/g extract. Hydroxytyrosol, verbascoside, luteolin 7-*O*-glucoside, and luteolin 4′-*O*-glucoside were also present in noticeable quantities. Genotypes differed to the greatest extent in the content of verbascoside (0.45–21.07 mg/g extract). The content of hydroxytyrosol ranged from 1.33 to 4.03 mg/g extract, and the aforementioned luteolin glucosides were present at 1.58–8.67 mg/g extract. The total phenolic content (TPC), DPPH^•^ and ABTS^•+^ scavenging activities, ferric reducing antioxidant power (FRAP), and ability to inhibit the oxidation of β-carotene-linoleic acid emulsion also varied significantly among genotypes. A hierarchical cluster analysis enabled the division of genotypes into three clusters with similarity above 60% in each group. GGE biplot analysis showed olive genotypes variability with respect to phenolic compound contents and antioxidant activities. Significant correlations among TPC, FRAP, the values of both radical scavenging assays, and the content of oleuropein were found. The contents of 7-*O*-glucoside and 4′-*O*-glucoside correlated with TPC, TEAC, FRAP, and the results of the emulsion oxidation assay.

## 1. Introduction

The evergreen olive tree (*Olea europaea* L.) is native to coastal Mediterranean areas and is one of the oldest crops in this region. Large amounts of by-products are generated by olive oil production processes, including olive leaves [1,2]. This cheap agro-industrial material is generally used as animal feed or energy biomass, but recently, interest in the potential use of olive leaves and olive leaf extracts in the pharmaceutical, food, and cosmetics industries is growing [2,3,4,5].

The biological activities of olive leaf compounds have been reported. These compounds have cholesterol lowering effects, antiviral and antibacterial activity against a wide range of microorganisms, radioprotective effect, in vitro and in vivo antioxidant activity, and antiproliferative effect against cancer and endothelial cells [6]. The potential of olive leaves in the prevention of hypertension, cardiovascular and neurological diseases, diabetes, and hyperlipidaemia has been shown [5]. The biological activity of the olive leaves allows them to be considered a functional food ingredient. The olive leaf components can also extend the shelf life of food products by limiting lipid oxidation or antimicrobial effects [2]. Therefore, several lipid-rich foods or refined oils enriched with olive leaf extracts showed remarkable oxidative stability. The antioxidant, antimicrobial, and anti-inflammatory properties of olive leaf extracts can impart usefulness as an ingredient in skin care products and cosmetics [4].

High-added-value constituents of olive leaf by-product are phenolic compounds [2]. These bioactive compounds with potential technological functions constitute approximately 2.5% of olive leaves [1] and can be extracted in good yields using conventional solvent extraction techniques or modern methods, e.g., ultrasound-assisted, microwave-assisted, supercritical fluid extraction, and pressurised liquid extraction [5]. The major phenolic constituent of olive leaves is oleuropein. This ester of hydroxytyrosol and elenolic acid glucoside is classified as a secoiridoids, a group of compounds present exclusively in plants belonging to the *Oleaceae* family [7]. In addition to secoiridoids, in olive leaves, there are significant quantities of phenylethanoids, such as tyrosol and hydroxytyrosol, as well as flavonoids represented by flavonols (primarily quercetin and isorhamnetin and their derivatives) and flavones (primarily apigenin and luteolin and their derivatives) [6]. The presence of phenolic acids and their derivatives was also noted [8]. Many phenolic compounds occurring in olive leaves had significant radical scavenging activity [9,10]. Additionally, synergistic behaviour among phenolic compounds in the olive leaf extract was observed [9]. The Trolox equivalent antioxidant capacity (TEAC) of the whole extract was higher with respect to the theoretical value obtained from the TEAC of individual phenolic compounds. 

It is well-known that various biotic and abiotic factors affect the quantitative and qualitative composition of phenolic compounds of natural materials and hence biological activity of plants/extracts. Olive leaves are not the exception in this respect [6]. One of the most important factors differentiating the profile of phenolic compounds and the antioxidant activity of olive leaves is the genotype of olive trees [11,12,13,14,15]. The content of major phenolic compounds of olive leaves, especially oleuropein, can be used as chemotaxonomic markers [6]. The use of various statistical models has enabled discrimination among cultivars [8,11,16].

The aim of this study was to comparatively analyse the phenolic compound profiles and antioxidant activities of the aqueous-methanolic extracts of *O. europaea* leaves sampled in genotypes grown in Turkey. Chemometric techniques, including hierarchical cluster and GGE biplot analyses, were applied to evaluate the genotypic variation and determining the most convenient genotypes with regard to antioxidant activity and phenolic compound content. To the best of our knowledge, the analysed genotypes were compared for the first time regarding their phenolic compound profiles and antioxidant activities.

## 2. Results and Discussion

### 2.1. Extraction Yield and Total Phenolic Content

The yield of olive leaf extracts varied from 24.46 to 29.87% (Table 1). The highest values were noted for ‘Ayvalik’, ‘Esek Zeytini’, and ‘Ascolana’ genotypes. In turn, ‘Uslu’ and ‘Saurani’ gave lower yields of extract. The total phenolic content (TPC) of olive leaf extracts ranged from 110 mg GAE/g to 268 mg GAE/g (Table 1) and decreased in the following order of genotypes: ‘Esek Zeytini’ > ‘Ayvalik’ = ‘Ascolan’ > ‘Kilis Yaglik’ > ‘Memecik’ = ‘Cekiste’ > ‘Gemlik’ = ‘Saurani’ > ‘Uslu’. The hot-water extracts obtained from leaves of the same genotypes were characterised by 1.4- to 2.6-fold lower TPC, but as in our research, ‘Esek Zeytini’ and ‘Uslu’ extracts were the most and least abundant in phenolic compounds, respectively [13]. Herrero et al. [17] determined a lower TPC of extracts prepared using pressurised liquid (water or ethanol) extraction (26.2–58.7 mg GAE/g). In turn, the TPC found in our study was in accordance with results determined for the methanolic extract of tree olive leaves from natural habitats and cultivated conditions: 127.18–314.69 mg GA/g [18] and for two cultivar leaf extracts obtained with different solvents, i.e., water, water-methanol (1:1, *v*/*v*), water-ethanol (1:1, *v*/*v*): 230.15–241.60 mg GAE/g [19]. When the yield of extraction was considered, the TPC of olive leaf of Turkish genotypes in the present study (27.0–79.7 mg GAE/g dry leaves, data not show) was similar to that noted for Spanish (52.2–60.64 mg/g dry weight of leaves) and Italian (40.9–66.6 mg GAE/g dry leaves) cultivars [14,20].

### 2.2. Identification and Quantification of Phenolic Compounds

**Figure 1:**
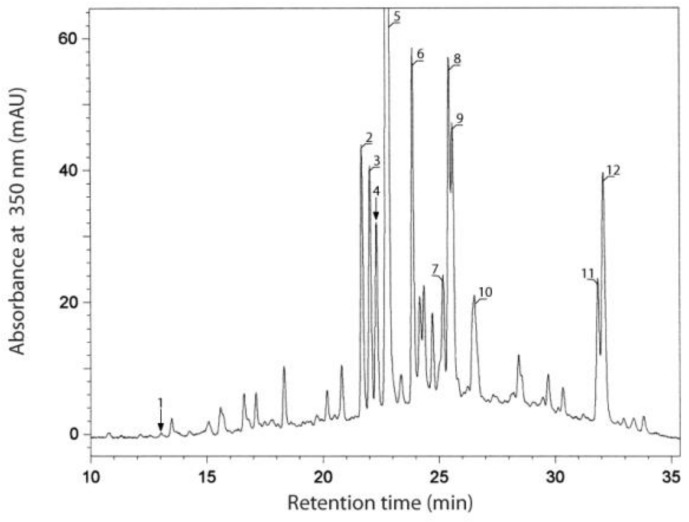
High-performance liquid chromatography (HPLC) separation of phenolic compounds of olive leaf extract. (**1**) hydroxytyrosol; (**2**) luteolin glycoside 1; (**3**) verbascoside; (**4**) luteolin glycoside 2; (**5**) luteolin 7-*O*-glucoside; (**6**) apigenin glycoside; (**7**) apigenin 7-*O*-glucoside; (**8**) luteolin 4′-*O*-glucoside; (**9**) luteolin glycoside 3; (**10**) oleuropein; (**11**) luteolin glycoside 4; (**12**) quercetin.

The HPLC separation of the phenolic compounds of olive leaf extract is shown in Figure 1. The compounds corresponding to peaks 1–12 were identified. They were detected in the extracts of all genotypes. The results of quantitative analysis of these compounds are presented in Table 2. Peak with retention time at 12.9 min, which was very small on the chromatogram recorded at 350 nm (Figure 1), originated from the compound with the maximum absorption of UV spectrum at 226 and 279 nm (data not shown). Based on these data and on comparison with the standard, the compound **1** was identified as hydroxytyrosol. This phenolic compound and its derivatives (glucosides in particular) were previously determined in olive leaves [8,15,21].

The content of hydroxytyrosol in extracts ranged from 1.33 mg/g (‘Uslu’) to 4.03 mg/g (‘Esek Zeytini’) (Table 2). Ortega–García and Peragón [12] reported greater variation in its content in olive leaves of certain Spanish cultivars. In turn, hydroxytyrosol content determined in a ‘Moraiolo’ olive leaf extract was within the range noted in our study [22].

Compound **10** was identified as oleuropein by comparison of its chromatographic and spectroscopic data to a standard. In the literature, the presence of oleuropein in olive leaves has been described frequently [9,15,23]. In our study, oleuropein appeared as a major phenolic component of the olive leaf extracts, although its content differed over a wide range among genotypes (Table 2). Oleuropein was the most abundant in ‘Esek Zeytini’ (98.0 mg/g) followed by ‘Ascolana’ (57.6 mg/g) extracts. The lowest content of oleuropein was determined in ‘Gemlik’, ‘Uslu’, and ‘Saurani’ extracts, at 21.0–23.1 mg/g, which did not differ statistically (*p* ≥ 0.05). The results are in accordance with these noted by Goldsmith et al. [19] for aqueous methanol and ethanol (50%, *v*/*v*) leaf extracts from olive tree cultivars growing in Australia. In addition, methanolic extracts from ‘Koroneiki’ and ‘Chetoui’ contained oleuropein at a similar level of approximately 14–90 mg/g [15]. It has been commonly reported that oleuropein is a major phenolic compound of olive leaves [9,22,24], although in several studies, other phenolic compounds have been found to prevail [8,25].

Verbascoside (**3**) was identified in extracts based on a reference substance. This glycosylated conjugate of caffeic acid and hydroxytyrosol was the second compound previously found in olive leaves [9,14,23]. The related molecules (verbascoside isomers, hydroxyverbascoside, metoxyverbascoside) were also detected in olive leaves using HPLC-MS/MS techniques [15,24]. The highest content of verbascoside was determined in ‘Kilis Yaglik’ extract (21.07 mg/g), but ‘Ayvalik’ and ‘Cekiste’ extracts contained as dramatic as 29.3- and 46.8-fold lower amounts of this compound, respectively (Table 2). In addition to genotypic differences, other factors such as sampling time, leaf age, and growing conditions affected the content of verbascoside in olive leaves [6,11]. In our study, some of these factors were excluded: Trees were grown under the same soil and climatic conditions and the leaves were collected within one month. Ryan et al. [7] reported that the partial degradation of oleuropein is responsible for the formation of verbascoside in olive peel and pulp. However, this observation did not apply to the leaf extracts of analysed genotypes. A statistically significant correlation between the content of verbascoside and oleuropein was not found (data not shown).

Compounds **2**, **4**–**9**, and **11**–**12** were classified as flavonoids belonging to the subclass of flavones and flavonols. Among flavones, luteolin 7-*O*-glucoside (**5**), apigenin 7-*O*-glucoside (**7**), and luteolin 4′-*O*-glucoside (**8**) were identified by comparison to standards. The structures of compounds **2**, **4**, **6**, **9**, and **11** were not full identified, but were tentatively included to luteolin glycosides (**2**, **4**, **9**, and **11**) and apigenin glycoside (**6**) based on shorter retention times than corresponding aglycons and the similarity of the shape and maxima of UV spectra to that of the aglycon [26]. The presence of several glycosides of luteolin and apigenin in olive leaves has been described in the literature [15,21,25]. In addition to luteolin 7-*O*-glucoside, apigenin 7-*O*-glucoside and luteolin 4′-*O*-glucoside, luteolin glucoside isomers, luteolin diglucoside isomers, luteolin 7-*O*-rutinoside and its isomers, and apigenin 7-*O*-rutinoside were noted. The luteolin and apigenin were not detected in our study, although both compounds were previously determined in olive leaves [17,25]. Luteolin 7-*O*-glucoside was the main flavone identified in the extracts of all genotypes (Table 1). The amount of luteolin 4′-*O*-glucoside and luteolin glycoside 3 was noted in ranges 1.58–3.90 and 1.27–3.61 mg/g, respectively. In turn, the content of apigenin 7-*O*-glucoside, luteolin glycoside 2 and luteolin glycoside 4 did not exceed 1 mg/g of the extract of any genotype. The luteolin 7-*O*-glucoside was previously reported as the dominant flavonoid of olive leaf extracts [17,27]. The extracts of ‘Kilis Yaglik’ and ‘Ascolana’ were the richest source of flavones, and ‘Uslu’ contained the lowest amount of these compounds (Table 2). However, the genotypic differences in the content of flavonoids were smaller than the abovementioned variations in the content of verbascoside and oleuropein. 

Compound **12** represented the subclass of flavonols. It was identified as quercetin by comparison of its chromatographic and spectroscopic data to a reference substance. Its content was low in olive leaf extracts (Table 2), consistent with literature data [17].

### 2.3. Antioxidant Activity of Olive Leaf Extracts

The olive leaf extracts were evaluated for their DPPH^•^ and ABTS^•+^ scavenging activities, for their abilities to reduce ferric ions and to inhibit the oxidation of a model emulsion with β-carotene and linoleic acid.

Antiradical activity against ABTS^•+^ expressed as TEAC is presented in Table 1. Olive leaf extracts of ‘Esek Zeytini’ and ‘Kilis Yaglik’ genotypes showed the highest activity with values that did not differ statistically (*p* ≥ 0.05)—1.01 mmol Trolox/g. The lowest ABTS^•+^ scavenging activity was exhibited by ‘Uslu’ extract (0.70 mmol Trolox/g). A similar trend was observed for ferric-reducing antioxidant power (FRAP) (Table 1). Again, extracts of ‘Esek Zeytini’ and ‘Uslu’ characterised the highest (2.12 mmol Fe^2+^/g) and lowest (1.04 mmol Fe^2+^/g) values, respectively. The ability by other genotypes to reduce ferric ions decreased in the order: ‘Esek Zeytini’ > ‘Kilis Yaglik’> ‘Ayvalik’ = ‘Ascolana’ > ‘Memecik’ > ‘Cekiste’ = ‘Gemlik’ > ’Saurani’ > ‘Uslu’. The two-fold differences in FRAP values and 1.5-fold differences in TEAC obtained for olive leaf extracts from Turkish genotypes (Table 1) were similar to those noted for cultivars growing in other countries, that is, Greece and Italy [20,28]. The significant correlation (*p* < 0.01) between the FRAP and TEAC of extracts of olive leaf genotypes with a correlation coefficient r = 0.789 was found (Table 3). The results of both antioxidant assays were also strongly correlated with TPC (Table 3). Linear correlations between TPC, TEAC, and FRAP were previously noted in the literature for samples of various cultivars of olive leaves [28] and other plant materials [29,30]. Benavente–García et al. [9] determined the ABTS^•+^ scavenging activity of pure compounds typical of olive leaves. TEAC of hydroxytyrosol was 1.57 mM. Activities of oleuropein and luteolin 7-*O*-glucoside were approximately 50% lower. In turn, TEAC of verbascoside and apigenin 7-*O*-glucoside was 1.02 and 0.42 mM. Considering this information and the content of these compounds in the analysed extracts (Table 2), it can be assumed that apart from apigenin 7-*O*-glucoside, the remaining compounds could contribute to the ABTS^•+^ scavenging activity of olive leaf extracts. Indeed, the contents of oleuropein and luteolin 7-*O*-glucoside were significantly correlated with TEAC (Table 3). Additionally, significant correlations were found between contents of these compounds and FRAP and TPC. TEAC, FRAP and TPC were also correlated with the content of luteolin 4′-*O*-glucoside (Table 3), although, due to its chemical structure, this compound is a less efficient radical scavenger than luteolin and its 7-*O*-glucoside [21].

**Figure 2:**
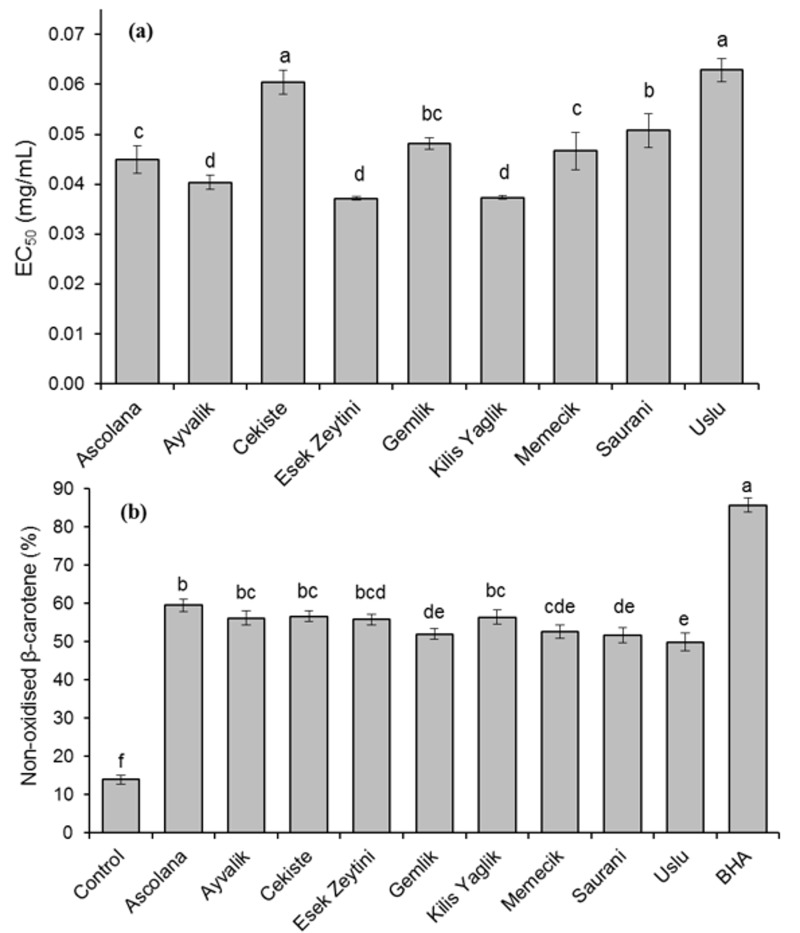
2,2-Diphenyl-1-picrylhydrazyl (DPPH) radical scavenging activity (**a**) and antioxidant activity in the β-carotene-linoleic acid emulsion (**b**) of olive leaf extracts. Data are expressed as mean ± standard deviation (*n* = 4) for extract of each genotype. Bars having different letters differ significantly (*p* < 0.05).

DPPH^•^ scavenging activity of olive leaf extracts is shown in Figure 2a. The highest antiradical activity towards DPPH^•^ was noted for ‘Esek Zeytini’ and ‘Kilis Yaglik’. The EC_50_ of extracts of these genotypes and additionally of ‘Ayvalik’ did not differ significantly from each other (*p* ≥ 0.05) and amounted to 0.037–0.040 mg/mL. In turn, ‘Cekiste’ and ‘Uslu’ had the highest EC_50_ (0.060–0.063 mg/mL). Stanković et al. [18] reported comparable DPPH^•^ scavenging activity of extracts of olive leaves from Tunisia, Malta, and Montenegro but higher IC_50_ values for French and Serbian samples, that is, 113.30 and 94.39 µg/mL, respectively. In our study, the EC_50_ values of analysed genotypes significantly correlated with TPC (r = −0.824; *p* < 0.01) and with results of ABTS assay (r = −0.676, *p* < 0.05) and FRAP assay (r = −0.873, *p* < 0.01) (Table 3). A strong correlation between phenolic content determined HPLC method and DPPH^•^ scavenging activity with notably high correlation coefficient (r = −0.953, *p* < 0.05) was previously found for the leaves of olive trees cultivars growing in Spain [14]. Goulas et al. [21] established that secoiridoids (primarily oleuropein) were responsible for 15–51% of the DPPH^•^ scavenging activity of olive leaf extracts. The contribution of hydroxytyrosol and flavonoids (primarily luteolin 7-*O*-glucoside) to the total activity was also noticeable (up to 32% and 27%, respectively). Verbascoside comprised a smaller share of the overall antiradical activity towards DPPH^•^, 3–18%. However, the response of individual compounds highly varied with olive cultivar and sampling period. Our results showed that only the content of oleuropein correlated with EC_50_ values (Table 3). The contribution other compounds of olive leaf extract to DPPH^•^ scavenging activity was not so directly related to their content by genotypes.

The antioxidant activity of olive leaf extracts in the β-carotene-linoleic acid emulsion is presented in Figure 2b. Notably, the variation of extracts obtained from different genotypes in terms their ability to inhibit the emulsion oxidation was lower than observed in other assays. After 180 min of oxidation, 49.9% to 59.5% of β-carotene remained non-oxidised. All extracts had lower antioxidant activity than synthetic antioxidant BHA. A strong correlation between the antioxidant activity of olive leaf extracts in the β-carotene-linoleic acid emulsion and the content of some luteolin derivatives in extracts (luteolin 7-*O*-glucoside, luteolin 4′-*O*-glucoside, luteolin glycoside 3) was found (Table 3). The correlation with oleuropein content was statistically nonsignificant (*p* = 0.082).

### 2.4. Chemometric Analysis

**Figure 3:**
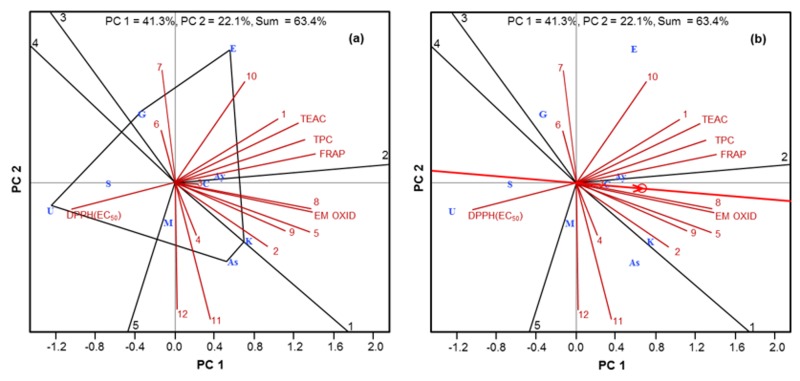
“Which-won-where/what” (**a**) and “average tester coordination” (**b**) views of GGE biplot. (**1**) hydroxytyrosol; (**2**) luteolin glycoside 1; (**3**) verbascoside; (**4**) luteolin glycoside 2; (**5**) luteolin 7-*O*-glucoside; (**6**) apigenin glycoside; (**7**) apigenin 7-*O*-glucoside; (**8**) luteolin 4′-*O*-glucoside; (**9**) luteolin glycoside 3; (**10**) oleuropein; (**11**) luteolin glycoside 4; (**12**) quercetin. As: ‘Ascolona’; Ay: ‘Ayvalik’; C: ‘Cekiste’; E: ‘Esek Zeytini’; G: ‘Gemlik’; K: ‘Kilis Yaglik’; M: ‘Memecik’; S: ‘Saurani’; U: ‘Uslu’.

The effects of genotypes on the phenolic compound distribution and antioxidant activity of nine olive genotypes were compared using GGE biplot analysis (Figure 3). The data obtained for the 12 phenolic compounds, four antioxidant assays, and TPC were subjected to analysis. The GGE biplot “which-won-where/what” shows a polygon view with some genotypes as vertices [31]. Perpendicular lines are drawn for each side of the polygon, and the biplot was divided into sectors. The vertex genotypes are the most responsive and are either best or poorest for one or all characteristics in each sector. In our study, five genotypes, ‘Ascolona’, ‘Kilis Yaglik’ ‘Esek Zeytini’, ‘Gemlik’, and ‘Uslu’ were located at a vertex of the polygon, and five sectors were obtained (Figure 3a). ‘Uslu’ and ‘Saurani’ were in the same sector and ‘Uslu’ was distinct from the other genotypes, given the high EC_50_ value of the DPPH assay. ‘Gemlik’ and ‘Esek Zeytini’ were best for the most characteristics: FRAP, TPC, TEAC and content of hydroxytyrosol, oleuropein, apigenin 7-*O*-glucoside, and apigenin glycoside. ‘Esek Zeytini’ had the highest vector in its respective direction. The vector length and direction represents the extension of genotype response to the treatments [32]. ‘Kilis Yaglik’ differed from other vertex genotypes with respect to luteolin glycosides 1 and 3, luteolin 7-*O*-glucoside, luteolin 4′-*O*-glucoside, verbascoside, and the ability to inhibit emulsion oxidation. The ‘Ascolona’ and ‘Memecik’ fall in the same sector and were best for quercetin and luteolin glycosides 2 and 4.

The “average tester coordination” view of the GGE biplot is presented in Figure 3b. This graph visualises the interrelationship among characteristics. The close positive associations between TPC, TEAC, FRAP, and the content of hydroxytyrosol and oleuropein are shown. Moreover, antioxidant activity in the emulsion system were strongly related to luteolin 4′-*O*-glucoside, luteolin 7-*O*-glucoside and luteolin glycosides 1 and 3, but poorly related to oleuropein. The last two observations agreed well with the results of the correlation analysis (Table 3) with the exception of hydroxytyrosol in the first case and luteolin glycoside 1 in the second. 

**Figure 4:**
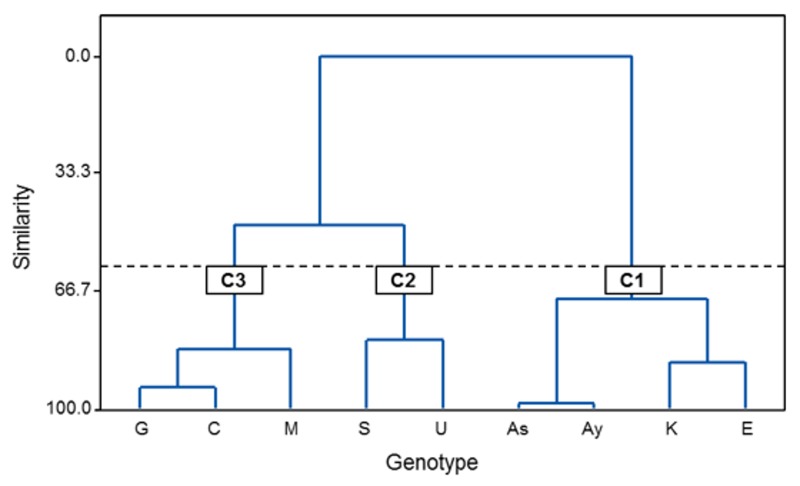
Dendrogram of hierarchical cluster analysis of olive genotypes for data of phenolic compound contents and antioxidant activity of olive leaf extracts. As: ‘Ascolona’; Ay: ‘Ayvalik’; C: ‘Cekiste’; E: ‘Esek Zeytini’; G: ‘Gemlik’; K: ‘Kilis Yaglik’; M: ‘Memecik’; S: ‘Saurani’; U: ‘Uslu’.

A hierarchical cluster analysis of olive genotypes was performed using data from phenolic compound contents and antioxidant activities. Three distinct clusters (C1–C3) were formed with similarity greater than 60% in each group (Figure 4). The mean values of variables for clusters are present in Table 4. The C1 included four genotypes, ‘Ascolana’, ‘Ayvalik’, ‘Kilis Yaglik’, and ‘Esek Zeytini’, associated with a high TPC and content of hydroxytyrosol, verbascoside, oleuropein and luteolin derivatives (luteolin 7-*O*-glucoside, luteolin 4′-*O*-glucoside, luteolin glycosides 2 and 4), as well as with high antioxidant activity, which may be explained by the presence of the mentioned compounds. C2 consisted of ‘Saurani’ and ‘Uslu’ and had the lowest antioxidant activities, TPC, and content of most individual phenolic compounds. The similarity between genotypes and their differentiation from others was consistent with the GGE biplot (Figure 3). Three genotypes (‘Gemlik’, ‘Memecik’ and ‘Cekiste’) were included in C3 (Figure 4). This group was distinguished by the highest content of hydroxytyrosol, apigenin derivatives and two luteolin glycosides, with fairly high TEAC and low DPPH^•^ scavenging activity. Previously, hierarchical clustering based on phenolic compound contents and the antioxidant activities of leaves was successfully used to classify cultivars of blueberry or various spices [33,34].

## 3. Materials and Methods

### 3.1. Plant Material

Leaves from olive trees (*Olea europaea* L.), nine genotypes, were obtained from the Olive Research Institute (Izmir, Turkey). The trees of each genotype were grown under the same soil and climatic conditions at the Station of Olive Growing in Bornova. The leaves of ‘Ayvalik’, ‘Cekiste’, ‘Esek Zeytini’, ‘Gemlik’, ‘Kilis Yaglik’, ‘Memecik’, ‘Saurani’, and ‘Uslu’ genotypes, which originated from Turkey, and of ‘Ascolana’, originating from Italy, were collected in May. Fresh leaves were transported to the laboratory, air-dried under ambient temperature, and finally pulverised in a mortar to particles with sizes < 0.8 mm.

### 3.2. Chemicals and Reagents

Folin-Ciocalteu’s phenol reagent, gallic acid, 2,2-diphenyl-1-picrylhydrazyl (DPPH), 2,2′-azinobis-(3-ethylbenzothiazoline-6-sulphonic acid) (ABTS), 2,4,6-tri(2-pyridyl)-*s*-triazine (TPTZ), 6-hydroxy-2,5,7,8-tetramethyl-chroman-2-carboxylic acid (Trolox), β-carotene, linoleic acid, butylated hydroxyanisole (BHA), trifluoroacetic acid (TFA) and quercetin were obtained from Sigma-Aldrich (St. Louis, MO, USA). Hydroxytyrosol, verbascoside, oleuropein, luteolin 7-*O*-glucoside, luteolin 4’-*O*-glucoside and apigenin 7-*O*-glucoside were purchased from Extrasynthese S.A. (Genay, France) Solvents and other chemicals, if not otherwise specified, were acquired from Avantor Performance Materials (Gliwice, Poland).

### 3.3. Extract Preparation

Powdered olive leaves were suspended in methanol-water (4:1, *v*/*v*) solution in a 1:10 (*v*/*w*) ratio of sample to extractant [25]. The bottles with suspensions were placed in a shaking water bath (SW22, Julabo, Seelbach, Germany) heated to 65 °C. Extraction was performed three times for 15 min. Filtrates obtained after each step of the process were combined, and organic solvent was evaporated using a Rotavapor R-200 (Büchi Labortechnik, Flawil, Switzerland). The aqueous residue was lyophilised (Lyph Lock 6 freeze-dry system, Labconco, Kansas City, MO, USA).

### 3.4. Determination of Total Phenolic Compound Content

The TPC of olive leaf extracts was determined by reaction with Folin-Ciocalteu’s phenol reagent and absorbance measurement at 725 nm (DU-7500 spectrophotometer, Beckman Instruments, Brea, CA, USA) [35]. The TPC was expressed as mg gallic acid equivalents (GAE) per g of extract.

### 3.5. Phenolic Compounds Analysis

The phenolic compounds of extracts were separated using a Shimadzu HPLC system (Kyoto, Japan) consisting of two LC-10AD_Vp_ pumps, an SCL-10A_Vp_ system controller, and an SPD-M10A_Vp_ photodiode array detector (PAD). A Luna C18 column (250 × 4.6 mm, 5 μm, Phenomenex, Torrance, CA, USA) was connected to the system. The mobile phase consisted of acetonitrile-water-TFA (5:94.9:0.1, *v*/*v*/*v*) [solvent A] and acetonitrile-TFA (99.9:0.1, *v*/*v*) [solvent B] and was injected onto the column with a flow rate of 1 mL/min in a gradient system from 5 to 40% of solvent B from 0–40 min. The injection volume was 20 μL of extract solution in methanol (10 mg/mL). The PDA scanned over a wavelength range of 200 to 400 nm. The individual phenolic compounds were identified based on comparison of their retention times and UV spectra with corresponding standards. The calibration curves of standards were used to quantify compounds. The hydroxytyrosol and oleuropein were determined at 280 nm, verbascoside at 320 nm and flavonoids at 350 nm.

### 3.6. Determination of Trolox Equivalent Antioxidant Capacity

An ABTS assay was conducted to determine the TEAC of the extracts. ABTS^•+^ was generated, and the reaction was performed exactly according to the procedure of Re et al. [36]. The absorbance of reaction mixtures was measured at 734 nm. The results were expressed as mmol Trolox equivalents (TE) per g of extract.

### 3.7. Determination of DPPH Radical Scavenging Activity

The scavenging activity of olive leaf extracts towards DPPH^•^ was determined according to the method described by Brand-Williams et al. [37]. Briefly, the portions of 2 mL of methanol were vortexed with methanolic solutions of DPPH (0.25 mL, 1 mM) and extracts (0.1 mL, 0.5–2.5 mg/mL). The reaction was conducted in the dark for 20 min, and the absorbance was measured at 517 nm. The curves of percent of absorbance versus extract content in the reaction mixture were plotted. EC_50_ values, defined as the concentration of extract needed to scavenge 50% of the initial DPPH^•^, were estimated from the plots.

### 3.8. Determination of Ferric-Reducing Antioxidant Power

The Benzie and Strain [38] method was used to determine FRAP of extracts. The FRAP reagent was prepared by mixing 10 mM TPTZ in 40 mM HCl (6 mL) with 300 mM acetate buffer at pH 3.6 (60 mL) and 20 mM ferric chloride (6 mL). Next, 75 μL of the extract solution and 225 μL of deionised water were added to 2.25 mL of FRAP reagent. The absorbance was measured at 593 nm after incubation of mixtures at 37 °C for 30 min. The results were expressed as mmol Fe^2+^ equivalents per g of extract based on the calibration curve prepared from ferrous sulphate.

### 3.9. β-Carotene-Linoleic Acid Emulsion Oxidation

The antioxidant activity of olive leaf extracts was evaluated using a β-carotene-linoleic acid model system [39]. The emulsion of linoleic acid and β-carotene in water was stabilised with Tween 40. The emulsion was oxidised in a 96-well plate [29]. Portions of the emulsion (250 µL) were vortexed with 20 μL of extract solution (1 mg/mL) or BHA (0.5 mg/mL). Methanol was added to the control sample. The plate was placed in an Infinite M1000 microplate reader (Tecan, Männedorf, Switzerland) heated to 42 °C. The absorbance was measured at 470 nm at 15-min intervals. The results were expressed as the percentage of non-oxidised β-carotene after 180 min of reaction.

### 3.10. Statistical Analysis

At least three analytical replications were conducted for antioxidant activity assays and HPLC analyses. The analysis of variance (one-way ANOVA) was followed by the least significant difference (LSD) test. Differences were considered to be statistically significant when *p* < 0.05. Hierarchical cluster analysis based on Ward’s method [40] was performed. The statistical package of MSTAT-C software was used. A simple relationship between variables for genotypes were calculated as the Pearson correlation (GraphPad Prism software, GraphPad Software Inc., La Jolla, CA, USA). The GGE biplot analysis was performed according to Yan and Rajcan [31]. Graphs were generated using the software GGE Biplot Package.

## 4. Conclusions

The aqueous-methanolic extracts obtained from olive leaves of eight Turkish and one Italian genotypes contained compounds belong to the secoiridoids, phenylethanoids and flavonoids. In the present study, 12 compounds were identified and quantified. Oleuropein dominated in all extracts. Several genotypes were also good sources of hydroxytyrosol, verbascoside, luteolin 7-*O*-glucoside and luteolin 4′-*O*-glucoside. The content of verbascoside varied in the widest range among extracts. In addition to the content of analysed individual compounds, also TPC, DPPH^•^, and ABTS^•+^ scavenging activities, FRAP, and antioxidant activity in β-carotene-linoleic acid emulsion differed significantly among genotypes. Significant correlations were found between TPC and results of antioxidant assays. In the case of individual phenolic compounds, TPC, FRAP, and TEAC were correlated with the content of oleuropein when all genotypes were considered and associated with the content of hydroxytyrosol for part of genotypes. In turn, the ability to inhibit emulsion oxidation strongly correlated with the content of luteolin derivatives (luteolin 7-*O*-glucoside, luteolin 4′-*O*-glucoside and luteolin-glycoside 3). In general, the compounds mentioned above made the strongest contribution to the antioxidant activity of olive leaves. 

The hierarchical cluster and GGE biplot analyses also indicated that the profile of phenolic compounds of leaf extracts and their antioxidant activities had reference values for the classification of olive genotypes. Several genotypes had high TEAC, FRAP, and TPC; others were more active in the β-carotene-linoleic acid emulsion. In general, extracts of ‘Esek Zeytini’ and ‘Kilis Yaglik’ were the best in respect of phenolic compound content and antioxidant activity. Only a small number of genotypes (‘Saurani’ and ‘Uslu’) were characterised by lower activity and lower content of the primary compounds. These differences can determine the potential applications of leaves of appropriate olive genotypes as a source of bioactive compounds in food, cosmetics, and pharmaceutical products.

## Figures and Tables

**Table 1:** Extract yield, total phenolic content (TPC), Trolox equivalent antioxidant capacity (TEAC) and ferric-reducing antioxidant power (FRAP) of olive leaf extracts of different genotypes.

Genotype	Extract Yield (%)	TPC mg GAE/g)	TEAC (mmol TE/g)	FRAP (mmol Fe^2+^/g)
Ascolona	29.58	236 ± 4.8 ^b^	0.83 ± 0.03 ^e^	1.79 ± 0.037 ^c^
Ayvalik	29.87	242 ± 3.2 ^b^	0.98 ± 0.08 ^b^	1.78 ± 0.027 ^c^
Cekiste	25.84	206 ± 0.4 ^d^	0.96 ± 0.06 ^c^	1.48 ± 0.048 ^e^
Esek Zeytini	29.79	268 ± 3.0 ^a^	1.01 ± 0.04 ^a^	2.12 ± 0.016 ^a^
Gemlik	27.39	199 ± 1.2 ^e^	0.93 ± 0.04 ^d^	1.42 ± 0.024 ^e^
Kilis Yaglik	28.52	225 ± 8.1 ^c^	1.01 ± 0.08 ^a^	1.99 ± 0.069 ^b^
Memecik	27.11	209 ± 3.4 ^d^	0.84 ± 0.10 ^d^	1.60 ± 0.074 ^d^
Saurani	24.63	197 ± 1.7 ^e^	0.75 ± 0.05 ^f^	1.23 ± 0.052 ^f^
Uslu	24.46	110 ± 4.3 ^f^	0.70 ± 0.14 ^g^	1.04 ± 0.019 ^g^

Data are expressed as the mean ± standard deviation (*n* = 3) for extract of each genotype. Values in the same column having different letters differ significantly (*p* < 0.05). GAE: Gallic acid equivalents. TE: Trolox equivalents.

**Table 2:** Content of individual phenolic compounds in leaf extracts of different olive genotypes (mg/g).

No	Compound	Ascolana	Ayvalik	Cekiste	Esek Zeytini	Gemlik	Kilis Yaglik	Memecik	Saurani	Uslu
1	Hydroxytyrosol	2.32 ± 0.12 ^c^	1.96 ± 0.10 ^d^	3.38 ± 0.17 ^b^	4.03 ± 0.20 ^a^	2.52 ± 0.13 ^c^	2.04 ± 0.10 ^d^	2.10 ± 0.11 ^d^	2.44 ± 0.12 ^c^	1.33 ± 0.07 ^e^
2	Luteolin glycoside 1 *	1.15 ± 0.06 ^c^	1.15 ± 0.06 ^c^	1.83 ± 0.09 ^a^	0.76 ± 0.07 ^d^	0.87 ± 0.04 ^d^	1.89 ± 0.09 ^a^	1.48 ± 0.04 ^b^	0.89 ± 0.04 ^d^	0.45 ± 0.02 ^e^
3	Verbascoside	6.28 ± 0.24 ^c^	0.72 ± 1.05 ^f^	0.45 ± 0.12 ^f^	4.89 ± 0.74 ^d^	19.55 ± 0.98 ^b^	21.07 ± 0.20 ^a^	3.47 ± 0.17 ^e^	3.25 ± 0.31 ^e^	3.92 ± 0.16 ^de^
4	Luteolin glycoside 2 *	0.84 ± 0.04 ^a^	0.47 ± 0.02 ^c^	0.37 ± 0.02 ^d^	0.22 ± 0.01 ^ef^	0.63 ± 0.03 ^b^	0.51 ± 0.03 ^c^	0.20 ± 0.01 ^f^	0.67 ± 0.03 ^b^	0.25 ± 0.01 ^e^
5	Luteolin 7-*O*-glucoside	8.11 ± 0.41 ^a^	7.43 ± 0.37 ^b^	6.84 ± 0.34 ^b^	5.69 ± 0.28 ^c^	5.23 ± 0.26 ^cd^	8.67 ± 0.43 ^a^	4.96 ± 0.25 ^de^	4.50 ± 0.23 ^e^	3.23 ± 0.16 ^f^
6	Apigenin glycoside **	0.57 ± 0.03 ^ef^	0.64 ± 0.03 ^d^	0.88 ± 0.01 ^b^	0.54 ± 0.03 ^f^	1.48 ± 0.07 ^a^	0.42 ± 0.02 ^g^	0.77 ± 0.04 ^c^	0.63 ± 0.03 ^de^	0.38 ± 0.02 ^g^
7	Apigenin 7-*O*-glucoside	0.20 ± 0.01 ^d^	0.40 ± 0.02 ^b^	0.21 ± 0.01 ^d^	0.40 ± 0.02 ^b^	0.59 ± 0.03 ^a^	0.08 ± 0.04 ^e^	0.18 ± 0.01 ^d^	0.29 ± 0.01 ^c^	0.18 ± 0.01 ^e^
8	Luteolin 4′-*O*-glucoside	3.72 ± 0.19 ^a^	3.64 ± 0.18 ^ab^	3.57 ± 0.18 ^ab^	3.54 ± 0.18 ^ab^	2.08 ± 0.10 ^c^	3.90 ± 0.20 ^a^	3.39 ± 0.17 ^b^	1.58 ± 0.08 ^d^	1.89 ± 0.09 ^c^
9	Luteolin glycoside 3 ***	2.98 ± 0.15 ^b^	2.60 ± 0.13 ^c^	3.61 ± 0.18 ^a^	1.91 ± 0.10 ^e^	1.92 ± 0.10 ^e^	2.63 ± 0.13 ^c^	2.28 ± 0.11 ^d^	1.41 ± 0.07 ^f^	1.27 ± 0.06 ^f^
10	Oleuropein	57.6 ± 2.9 ^b^	46.3 ± 2.3 ^c^	38.5 ± 1.9 ^d^	98.0 ± 4.9 ^a^	23.1 ± 1.2 ^e^	44.7 ± 2.2 ^c^	38.2 ± 1.9 ^d^	21.0 ± 1.0 ^e^	22.2 ± 1.1 ^e^
11	Luteolin glycoside 4 *	0.69 ± 0.03 ^a^	0.54 ± 0.03 ^cd^	0.51 ± 0.02 ^de^	0.31 ± 0.02 ^f^	0.50 ± 0.02 ^de^	0.61 ± 0.03 ^b^	0.59 ± 0.03 ^bc^	0.49 ± 0.03 ^de^	0.47 ± 0.02 ^e^
12	Quercetin	1.92 ± 0.10 ^a^	1.07 ± 0.05 ^c^	0.63 ± 0.03 ^d^	0.43 ± 0.02 ^e^	0.47 ± 0.02 ^e^	1.36 ± 0.07 ^b^	1.86 ± 0.09 ^a^	1.24 ± 0.06 ^bc^	1.22 ± 0.06 ^bc^

Data are expressed as mean ± standard deviation (*n* = 3) for extract of each genotype. Values in the same row having different letters differ significantly (*p* < 0.05). * Expressed as luteolin 7-*O*-glucoside. ** Expressed as apigenin 7-*O*-glucoside. *** Expressed as luteolin 4′-*O*-glucoside.

**Table 3:** Pearson’s correlation coefficients (r) between total phenolic content (TPC), individual phenolic compound contents and antioxidant activities of olive leaf extracts of different genotypes.

	TPC	TEAC	FRAP	DPPH (EC_50_)	Emulsion Oxidation ^a^
Hydroxytyrosol	0.614	0.555	0.461	−0.225	0.369
Luteolin glycoside 1	0.359	0.518	0.395	−0.190	0.525
Verbascoside	0.043	0.303	0.218	−0.363	−0.018
Luteolin glycoside 2	0.179	0.107	−0.014	−0.148	0.352
Luteolin 7-*O*-glucoside	0.669 *	0.666 *	0.728 *	−0.570	0.903 **
Apigenin glycoside	0.040	0.201	−0.204	0.117	−0.186
Apigenin 7-*O*-glucoside	0.227	0.252	−0.014	−0.171	−0.186
Luteolin 4′-*O*-glucoside	0.689 *	0.699 *	0.833 **	−0.544	0.846 **
Luteolin glycoside 3	0.464	0.552	0.436	−0.095	0.806 **
Oleuropein	0.744 *	0.664 *	0.836 **	−0.674 *	0.608
Luteolin glycoside 4	−0.004	−0.149	0.024	−0.049	0.380
Quercetin	−0.104	−0.480	−0.024	−0.059	0.140
TPC	1	0.746*	0.885 **	−0.824 **	0.737 *
TEAC		1	0.789 **	−0.676*	0.582
FRAP			1	−0.873 **	0.748 *
DPPH (EC_50_)				1	−0.485

^a^ Non-oxidized β-carotene after 180 min of reaction. * Correlation is significant at *p* < 0.05. ** Correlation is significant at *p* < 0.01.

**Table 4:** Mean values of variables for genotype clusters obtained by hierarchical cluster analysis.

Cluster No.	1	2	3	4	5	6	7	8	9	10	11	12	TPC (mg GAE/g)	TEAC (mmol TE/g)	FRAP (mmol Fe^2+^/g)	DPPH EC_50_ (mg/mL)	Emulsion Oxidation (%)
	(mg/g)																
C1	2.69	1.24	14.02	0.51	7.48	0.54	0.27	3.70	2.53	61.63	0.54	1.20	242.83	0.96	1.92	0.107	56.98
C2	1.89	0.67	3.59	0.46	3.87	0.51	0.24	1.74	1.34	21.54	0.48	1.23	153.55	0.72	1.14	0.113	50.75
C3	2.67	1.39	7.82	0.40	5.68	1.04	0.33	3.01	2.60	33.29	0.53	0.99	204.69	0.91	1.50	0.118	53.73

(**1**) hydroxytyrosol; (**2**) luteolin glycoside 1; (**3**) verbascoside; (**4**) luteolin glycoside 2; (**5**) luteolin 7-*O*-glucoside; (**6**) apigenin glycoside; (**7**) apigenin 7-*O*-glucoside; (**8**) luteolin 4′-*O*-glucoside; (**9**) luteolin glycoside 3; (**10**) oleuropein; (**11**) luteolin glycoside 4; (**12**) quercetin; C1–C3: genotype clusters presented in Figure 4; GAE: gallic acid equivalents. TE: Trolox equivalents.
